# Multidisciplinary neoadjuvant management for potentially curable pancreatic cancer

**DOI:** 10.1002/cam4.444

**Published:** 2015-03-13

**Authors:** Neelam V Desai, Sarunas Sliesoraitis, Steven J Hughes, Jose G Trevino, Robert A Zlotecki, Alison M Ivey, Thomas J George

**Affiliations:** 1 Division of Hematology and Oncology, Department of Medicine, University of FloridaGainesville, Florida; 2 Department of Surgery, University of FloridaGainesville, Florida; 3 Department of Radiation Oncology, University of FloridaGainesville, Florida; 4 University of Florida Health Cancer CenterGainesville, Florida

**Keywords:** Chemotherapy, neoadjuvant, pancreatic cancer, personalized oncology, radiation, surgery

## Abstract

Pancreatic adenocarcinoma remains the fourth leading cause of cancer mortality in the U.S. Despite advances in surgical technique, radiotherapy technologies, and chemotherapeutics, the 5-year survival rate remains approximately 20% for the 15% of patients who are eligible for surgical resection. The majority of this group suffers metastatic recurrence. However, despite advances in therapies for patients with advanced pancreatic cancer, only surgery has consistently proven to improve long-term survival. Various combinations of chemotherapy, biologic-targeted therapy, and radiotherapy have been evaluated in different settings to improve outcomes. In this context, a neoadjuvant (preoperative) treatment strategy offers numerous potential benefits: (1) ensuring delivery of early, systemic therapy, (2) improving selection of patients for surgical therapy with truly localized disease, (3) potential downstaging of the neoplasm facilitating a negative margin resection in patients with locally advanced disease, and (4) providing a superior clinical trial mechanism capable of rapid assessment of the efficacy of novel therapeutics. This article reviews the recent trends in the management of pancreatic adenocarcinoma, with a particular emphasis on a multidisciplinary neoadjuvant approach to treatment.

## Background

Pancreatic adenocarcinoma is the fourth leading cause of cancer deaths in the United States 1. The annual incidence of pancreatic cancer is rising with approximately 46,420 new cases and nearly 39,590 patient deaths in 2014 2. Without any substantive improvement in curative therapies, it is anticipated to be the second leading cause of cancer deaths by 2030 3. Surgical resection is currently the only treatment option that offers the potential of long-term survival. However, only 20% of patients with pancreatic cancer are candidates for resection. Another 30–40% of patients have locally advanced or unresectable pancreatic cancer without measurable metastatic disease. For this group, chemotherapy with radiotherapy (chemoRT) was established as the standard of care over radiation or chemotherapy alone a few decades ago by the Gastrointestinal Tumor Study Group (GITSG) 4,5. For these patients, chemotherapy with radiation is palliative in nature with a median survival of 8–12 months and virtually no long-term survivors 6,7. Of the patients that present with resectable disease, surgical resection provides a 5-year survival of approximately 20%. This article focuses on the recent advances made in combined modality treatment of early stage resectable and borderline-resectable pancreatic adenocarcinoma with the goal of making a compelling case for a multidisciplinary, collaborative, and neoadjuvant approach for optimal outcomes. This strategy also facilitates an ideal clinical research platform capable of rapidly assessing the efficacy of novel therapeutic agents.

## Role of Adjuvant Therapy in Pancreatic Cancer

Despite improvements in surgical techniques that allow more patients to undergo successful R0 resection, the prognosis even for small tumors without nodal involvement remains poor due to progressive systemic disease. In an effort to improve long-term survival after surgical resection, adjuvant therapy has been studied in various combinations. In 1985, the GITSG conducted a trial that was one of the first to show the benefit of adjuvant therapy by demonstrating that 5-Fluorouracil (5-FU) combined with radiotherapy (RT) after surgical resection led to improved survival compared to observation (2-year survival 42% vs. 15%; *P* = 0.03) 8. The EORTC 40891 trial similarly compared 5-FU-based chemoRT to observation after surgical resection of pancreatic and periampullary adenocarcinoma with median overall survival (OS) of 24.5 versus 19 months (*P* = 0.21) 9. One of the major criticisms of the latter study was that it included periampullary adenocarcinomas, which have a better prognosis compared to pancreatic ductal adenocarcinomas. The rationale for adjuvant chemoRT was established with these early studies.

The role of adjuvant chemoRT was subsequently called into question in the European ESPAC-1 trial 10. Following surgical resection, patients were randomized to either receive 5-FU-based chemotherapy, 5-FU-based chemoRT, both or no treatment following surgical resection. Results were analyzed (in a two-by-two factorial design) based on groups having received chemotherapy or not and those having received chemoRT or not. Median OS in the chemotherapy group was 20.1 versus 15.5 months in the no chemotherapy group (*P* = 0.009). However, in the chemoRT analysis, the median OS was worse at 15.9 months compared to 17.9 months in patients who did not receive chemoRT (*P* = 0.05). This controversial trial is criticized for the lack of radiation quality control, use of outdated radiotherapy delivery techniques, no central review of radiographic response and poor compliance to subscribed treatment. Such limitations confound the ability to accurately and conclusively interpret the results. Nevertheless, the utilization of adjuvant chemoRT remains common in the United States with the benefit of this approach continuing to be actively investigated.

Subsequent European adjuvant clinical trials focused on the relative value of adjuvant chemotherapy rather than chemoRT. In the CONKO-1 trial, patients with resected pancreatic cancer were randomly assigned to either receive six cycles of gemcitabine or observation 11. The use of adjuvant gemcitabine resulted in significant gains in disease-free survival (DFS) from 6.7 to 13.4 months (*P* < 0.001) and a significant, albeit small, improvement in OS from 20.2 to 22.8 months (*P* = 0.01). Following this trial, the ESPAC-3 study compared adjuvant gemcitabine to 5-FU-based chemotherapy for 6 months 12. Median OS was not statistically different between the two treatment groups (23.6 vs. 23 months; *P* = NS). The overall rates of serious adverse events were significant reduced in the gemcitabine group (7.5%) compared to 5-FU-based treatment (7.5% vs. 14%; *P* < 0.001), with the toxicity profile also favoring gemcitabine with less stomatitis and diarrhea but more myelosuppression.

In the United States, the incorporation of adjuvant chemoRT continues to be investigated with trials designed to determine the optimal chemotherapy agents and sequencing for use in combination with radiotherapy. The RTOG 97-04 trial compared gemcitabine to 5-FU in a sequential combination of systemic chemotherapy (gemcitabine vs. 5-FU) for 3 weeks followed by 5-FU-based chemoRT and then 3 months of chemotherapy with the same previously used agent. Median OS was 20.5 months for gemcitabine versus 16.9 months for 5-FU (*P* = 0.09) 13. The above trials suggest that whether combined with radiation or not, gemcitabine may be a preferred agent in the adjuvant setting given a better side-effect profile; whereas 5-FU remains the next best option.

## Adjuvant Versus Neoadjuvant Debate

Despite the noted improvements in survival with the addition of adjuvant therapy, the 5-year OS still averages 20% in patients who undergo curative treatment, leaving significant opportunities for improvement. This has led to an increasing interest to incorporate chemotherapy and/or radiation therapy into the neoadjuvant setting. Neoadjuvant treatment may have several advantages over adjuvant therapy (Table1). First, as the vast majority of patients who undergo complete surgical resection still succumb to distant relapse, delivery of systemic treatment earlier in the disease course; particularly, when the anatomy and vasculature have not yet been disrupted by surgery, might lead to improved treatment effect. Second, almost 30–40% of postoperative patients do not receive any adjuvant therapy either secondary to surgical complications and delayed recovery or patient refusal 14. Third, tumor downstaging resulting from effective neoadjuvant treatment could lead to more effective R0 (complete) resections, which has been shown to be a predictor of survival 15. However, tumor downstaging requires accurate confirmation of the clinical stage prior to initiation of therapy, which is limited by the accuracy of current imaging modalities. Finally, the neoadjuvant platform is perhaps the most efficient *in vivo* model to test novel therapies as the treatment period is finite and pre/posttreatment tissue collection allows for a variety of molecular analyses to gain further insight into tumor biology and mechanisms of resistance. Opponents of neoadjuvant treatment voice concerns that the use of preoperative therapy can lead to a missed window of opportunity for surgical resection, which is the only potentially curative treatment. Such missed opportunities can result from progression of disease (typically distant metastases) or a decline in performance status from treatment toxicities or cancer cachexia. However, the issue of disease progression can also be seen as a paradoxical benefit given the morbidity of a major operative procedure such as pancreaticoduodenectomy in a patient with a biologically aggressive disease that might have likely relapsed soon after surgery. However, delaying surgical resection due to performance status decline from treatment side effects remains a legitimate concern. While no phase III trials exist, several retrospective series, prospective phase II, and systematic reviews have been published which provide some data with regards to neoadjuvant therapy outcomes.

**Table 1:** Potential advantages and disadvantages of neoadjuvant treatment in pancreatic adenocarcinoma

Neoadjuvant treatment	
Advantages	Disadvantages
Intact tumor vasculature not disrupted by surgery	Progression of disease during neoadjuvant treatment leading to missed window of opportunity for resection
Early treatment of micrometastatic disease	Toxicity from neoadjuvant treatment precluding definitive surgical resection
Ensures delivery of systemic treatment	Need tissue confirmation of neoplastic process
Improved RO resection rate; especially in borderline-resectable cases	
Ideal in vivo platform for research	

## Neoadjuvant Treatment of Pancreatic Cancer

The concept of incorporating neoadjuvant therapy into pancreatic cancer management started soon after early studies demonstrated a benefit of adjuvant treatment as compared to surgery alone. As early as 1980, Pipelich et al. showed that preoperative radiotherapy was not only feasible but allowed for downstaging of primary tumors and thus successful surgical resection 16. Since then, various combinations of chemotherapy, chemoRT or induction chemotherapy followed by chemoRT have been studied in relatively small phase I–II trials.

Early studies focused on 5-FU-based treatment combinations. The combination of 5-FU and mitomycin C (MMC) with external beam radiotherapy (EBRT) was particularly popular at that time. This was in part related to the widespread availability of the agents and their well-established role as potent radiosensitizers. Most of these trials consistently showed superior survival with the use of neoadjuvant treatment in resected patients as compared to historical controls who only received surgical resection. However, the direct impact of this treatment modality on resection rates is difficult to quantify for several reasons. First, the preoperative imaging quality during that time was limited in determining resectability. Second, even in cases where resectability status was based on laparotomy, there was institutional and surgeon variability with regards to expertise and definitions of resectability. While most of these studies demonstrated safety and feasibility, some showed survival comparable to that of studies involving adjuvant therapy. One such study by Hoffman et al. in 1995 included patients with both unresectable and resectable pancreatic cancers of adenocarcinoma and adenosquamous histology 17. Patients received preoperative 5-FU plus MMC with concurrent radiotherapy. The resection rate for all patients was 32% with resected patients having a reported median survival of 45 months; which is almost twice as long as reported in other studies. Promising as these results appear, it is hard to apply data from such small, single arm, single institution studies due to inherent selection bias and the heterogeneity of the study population.

In the decade following, gemcitabine-based neoadjuvant combinations gained popularity due to the positive findings reported by Burris and colleagues in the metastatic setting with regards to clinical benefit as well as a modest survival advantage 18. As a result of that latter study, gemcitabine received FDA approval and has become an established standard of care in advanced disease and in the adjuvant setting. The most frequently used neoadjuvant combinations were gemcitabine with or without an additional agent (including radiotherapy in some studies). The resection rate was noted to be variable depending on the initial resectability status of the patients enrolled. In the earlier trials, the overall resection rate after neoadjuvant therapy for patients who were deemed to have resectable disease upfront ranged from 50% to 70%. For those patients judged to be unresectable at the time of enrollment, the overall resection rate after neoadjuvant treatment ranged from 5% to 30%. More recent trials have shown an improved trend in both resection rates and survival for patients resected after preoperative treatment. In the modern era of studies, patients initially deemed resectable have resection rates in the 60–80% range with OS improving from 20 to 30 months for those patients receiving preoperative therapy. However, most of these studies were single institution or retrospective in design.

In an initial phase II trial from MD Anderson Cancer Center, 86 patients with resectable, histologically proven adenocarcinomas of pancreatic head or uncinate process were treated with neoadjuvant therapy 19. These patients underwent preoperative treatment with gemcitabine weekly for 7 weeks along with 30 Gy of EBRT over 2 weeks. The overall resection rate was 74%, (57/64 patients had R0 resections) with median survival of those patients undergoing resection noted to be 34 months. However, the majority of cases that relapsed did so with distant metastases. Therefore, in an attempt to improve the OS, Varadhachary and colleagues incorporated more systemic therapy by adding induction chemotherapy with gemcitabine plus cisplatin for four doses followed by chemoRT using gemcitabine weekly with 30 Gy EBRT 20. Of the 90 patients enrolled, 79 were able to complete neoadjuvant treatment. The overall resection rate for these 79 patients was 66% (51/52 patients had R0 resections) with median OS for those resected being 31 months. Memorial Sloan-Kettering Cancer Center treated 38 patients with gemcitabine with oxaliplatin for four cycles neoadjuvantly 21. Thirty-five patients (92%) completed neoadjuvant chemotherapy; 27 were ultimately resected (72%) and 23 (60.5%) were able to complete all planned treatments including additional adjuvant chemotherapy. Median OS was 27.2 months suggesting improvement in ability to complete the delivery of multimodality therapy. Additional prospective and retrospective neoadjuvant trials are summarized in Table2.

**Table 2:** Neoadjuvant therapy trials in pancreatic adenocarcinoma

Author/year	Location	Resectability (n)	Neoadjuvant therapy	Resection rate	Median OS unresected	Median OS resected
**Prospective studies**						
Weese JL 1990 33	Head (14), Body (1)	R = 15	5-FU + Mitomycin-C + EBRT	ORR = 60%	7 month	NR
				R0 = 60%		
Coia LR 1994 34	Pancreatic head/body (27) *Duodenum (4)*	R = 10	5-FU + Mitomycin-C + EBRT	ORR = 55% (48% panc) R0 = 49%	Pancreatic cancer only 15% 1-year	Pancreatic cancer only 60% 1-year
				UR = 21 (all panc)		
Hoffman JP 1995 17	Head/body/tail adenosquamous included	R = 21	5-FU + Mitomycin-C + EBRT	ORR = 32%	NS	45 month
		UR = 13		R0 = 29%		
Kamthan AG 1997 35	Head/body/tail	UR = 35	5-FU + Streptozocin + Cisplatin + EBRT → 5FU/leucovorin	ORR = 15%	All patients = 15 month	31 month
				R0 = NS		
Hoffman JP 1998 36	Head/body/tail	R = 53	5-FU + Mitomycin-C + EBRT	ORR – 45%	No surg – 5 month, Surg and no resection – 8 month	15 month
				R0 – 32%		
Staley CA 1996 37	Head	R = 39	5-FU + EBRT	ORR = 100%	NA	19 month
				R0 = 82%		
Pisters PW 1998 38	Head	R = 35	5-FU + rapid fractionation EBRT. Additional intraop RT only if PD	ORR = 57%	7 month	25 month
				R0 = 51%		
Bajetta E 1999 39	Head/body/tail	UR = 32	5-deoxyfluridine/leucovorin + EBRT	ORR = 16%	All patients = 9 month	NS
				R0 = 16%		
Snady H 2000 40	Head/body/tail	UR = 68	5-FU + Streptozocin + Cisplatin + EBRT	ORR = 29%	21 month	32 month
				R0 = 28%		
Wanebo HJ 2000 41	Head/body	R = 5	5FU + Cisplatin + EBRT	ORR = 64%	9 month	19 month
		UR = 6		R0 = 64%		
		Unk = 3				
Pipas JM 2001 42	Head/body/tail	UR = 21	Gemcitabine + EBRT (Phase I study)	ORR = 24%	NS	NS
				R0 = 24%		
Wolff RA 2001 43	Head	UR = 18	Gemcitabine + EBRT (Phase I study)	ORR = 5%	All patients = 6 month	NS
				R0 = 5%		
De Lange SM 2002 44	Head/body/tail	UR = 24	Gemcitabine + EBRT → Gemcitabine	ORR = 4%	All patients = 10 month	NS
				R0 = 4%		
Epelbaum R 2002 45	Head/body/tail	UR = 20	Gemcitabine → Gemcitabine + EBRT	ORR = 15%	All patients = 8 month	24 month
				R0 = 10%		
Pisters PW 2002 46	Head/uncinate process	R = 35	Paclitaxel + EBRT Additional intraop RT only if PD	ORR = 57%	All patients = 12 month	19 month
				R0 = 40%		
Al-Sukhun S 2003 47	Head/body/tail	UR = 20	Cisplatin + 5-FU + Cytarabine → Caffeine → 5-FU + EBRT	ORR = 15%	All patients = 13 month	24 month
				R0 = NS		
				R1 = NS		
Brunner TB 2003 48	Head/body/tail	UR = 30	Cisplatin + Gemcitabine + EBRT	ORR = 31%	11 month	18 month
				R0 = 28%		
Joensuu 2004 49	Head/body/tail	R = 28	Gemcitabine + EBRT	ORR = 71%	NS	NS
				R0 = 71%		
Moutardier V 2004 50	Head/body	R = 61	5-FU + Cisplatin + EBRT	ORR = 65%	All patients = 13 month	26.6 month
				R0 = 60%	UR = 8.6 month	
Magnino A 2005 51	Head/body/tail	UR = 23	Gemcitabine + EBRT	ORR = 26%	All patients = 14 month	20 month
				R0 = 22%	UR = 12 month	
Pipas JM 2005 52	Head/body	R = 4	Docetaxel + Gemcitabine → Gemcitabine + EBRT	ORR = 71%	All patients = 14 month	NS
		BR = 7		R0 = 54%		
		UR = 13				
Mornex F 2006 53	Head/body/tail	R = 41	5-FU + Cisplatin + EBRT	ORR = 63%	All patients = 9.4 month	11.7 month
				R0 = 51%		
Talamonti MS 2006 54	Head/body/tail	R = 20	Gemcitabine → Gemcitabine + EBRT	ORR = 85%	All patients = 18 month	26 month
				R0 = 80%		
Wilkowski R 2006 55	Head/body/tail	UR = 32	Gemcitabine + 5-FU + EBRT → Gemcitabine + Cisplatin	ORR = 18%	All patients = 13.6 month	16.4 month
				R0 = 12%		
Desai SP 2007 56	Head/body/tail/mets	R = 12	Gemcitabine + Oxaliplatin + EBRT	ORR = 16%	UR/metastatic = 9.1 month	20.8 month
		UR = 29		R0 = 16%		
		^*^metastatic = 3				
Palmer DH 2007 57	Head of pancreas by radiology	R = 50	Randomized phase II study: (A)Gemcitabine (*N* = 24) (B)Gemcitabine + Cisplatin (*N* = 26)	ORR = 38% in A and 70% in BR0 = 75% in both	All patients (A) = 9.9 month, (B) = 15.6 month	28.4 month
	Sp resection 3 pts had malignancy other than pancreatic adenoca					
Evans DB 2008 19	Head/uncinate process	R = 86	Gemcitabine + EBRT	ORR = 74%	All patients = 22.7 month	34 month
				R0 = 66%	UR = 7 month	
Heinrich S 2008 58	Head	R = 28	Gemcitabine + Cisplatin	ORR = 86%	NS	26.5 month
				R0/R1 = NS		
Le Scodan 2008 59	Head/body/tail	R = 41	5-FU + Cisplatin + EBRT	ORR = 63%	All patients = 9.4 month	11.7 month
				R0 = 51%		
Marti JL 2008 60	Head/body/tail	UR = 23	Gemcitabine + Cisplatin (*n* = 26) → Gemcitabine + Cisplatin + EBRT (*n* = 18)	ORR = 15%	All patients = 13 month	17 month
		BR = 3		R0 = 12%		
Small W 2008 61	Head/body/tail	R = 16	Gemcitabine + EBRT	ORR = 81% for R, 33% for BR, 7% for UR	All patients 1-year 73%	NS
		BR = 9				
		UR = 14				
Varadhachary GR 2008 20	Head/uncinate	R = 90	Gemcitabine + Cisplatin → Gemcitabine + EBRT	ORR = 58%	All patients 17.4 month, UR 10 month	31 month
				R0 = 56%		
Bjerregaard JK 2009 62	Head/body/tail	UR = 63	UFT + folinic acid + EBRT	ORR = 17%	8.8 month	46 month
				R0 = 17%		
Choi M 2010 63	Head/body/tail	UR = 20	Cisplatin + Cytarabine + Caffeine + 5-FU → 5-FU + EBRT	ORR = 15%	All patients = 13.7 month	24.3 month
				R0 = NS		
Laurent S 2009 64	Head/body/tail ^*^also had biliary cancer but outcomes separated	UR = 17	Gemcitabine + Oxaliplatin → Gemcitabine + Oxaliplatin + EBRT	ORR = 17%	All patients = 17 month	NS
				R0 = 17%		
Maximous DW 2009 65	Head/body/tail	R = 25	Gemcitabine + EBRT	ORR = 32%	All patients = 12 month	1-year = 87%
				R0 = NS	Unresected 1-year = 22%	
Turrini O 2010 66	Head/body/tail	R = 34	Docetaxel + EBRT	ORR = 50%	All patients = 15.5 month, unresected 11 month	32 month
				R0 = 50%		
Landry J 2010 67	Head/body/tail	R = 10	Randomized phase II: [A] Gemcitabine + EBRT (*n* = 10) [B] Gemcitabine + Cisplatin + 5-FU → 5-FU + EBRT (*n* = 11).	ORR, A = 30%, B = 18%	All patients, arm A = 19.4 month, arm B = 14.2 month	22 month in both A and B
	4 with “other” histology	UR = 8				
		? = 3				
			All patients got adjuvant Gemcitabine			
Sahora K 2010 68	Head/body/tail	UR = 18	Gemcitabine + Oxaliplatin	ORR = 39%	12 month	22 month
		BR = 15		R0 = 27%		
				R1 = 9%		
Lee JL 2012 69	Head/body/tail	BR = 18	Dose Dense Gemcitabine + Capecitabine	PR = 18.6%	13.1 month	23.1 month
		UR = 25		SD = 69.8%		
				R0 = 82% out of 17 resected		
				R1 = 18%		
Pipas JM 2012 70	Head/body/tail	R = 4	Cetuximab load then	ORR = 91% (PR = 30%)	10 month	24.3 month
		BR = 23	Cetuximab + Gemcitabine + EBRT			
		UR = 6		R0 = 92% out of 70% resected		
Satoi S 2012 71	Head/body/tail	R = 23	S1 + EBRT	ORR = 88% (PR = 18%)	NS	NS
		BR/UR = 7		R0 = 93%		
Chao YJ 2014 72	Head/body/tail	UR=41	Gemcitabine+5FU+Oxaliplatin+Thalidomide or Gemcitabine+5FU+Oxaliplatin+Sunitinib or Gemcitabine-based Chemoradiation	RR=51.2% (CR=5%, PR=46%)R0 = 31%, R1 = 5%, R2 = 2%	9 month	21 month
Golcher H 2014 73	Head	R = 73	Primary surgery or Gemcitabine + Cisplatin + EBRT	4 PR, 8 SD in treatment group	NR	18.9 versus 25 month
				R0 = 48 and 52%		
O’Reilly EM 2014 21	Head/body/tail	R = 38	Gemcitabine + Oxaliplatin	PR = 10.5%	27.2 month (all)	NS
				SD = 73.7%		
				R0/R1 = 77%		
**Retrospective studies**						
White RR 2001 74	Pancreatic head/body/tail	R = 53,	5-FU-based chemotherapy + EBRT	53% in R group, 19% in BR or UR	NS	Actuarial 2 year OS 32%
		BR or UR = 58	[5FU alone (n = 71), w mitomycin (n = 17), with cisplatin (n = 4), combination of 5Fu/mito/cis (n = 13), oral 5FU (n = 6)]			
Aristu J 2003 75	head/body/tail,	UR = 49	Chemotherapy + EBRT (one of 3 chemo):	ORR = 19%	10 month	22 month
			Cisplatin + 5FU			
			Cisplatin + 5FU +/− Paclitaxel			
			Gemcitabine + Docetaxel.			
			(23 UR pts got additional EBRT)			
Calvo FA 2004 76	Head/body	R = 15	Tegafur + EBRT	ORR = 60%	8 month (UR) 17 month (all)	23 month
				R0 = 46% overall		
Sa Cunha 2005 77	Head/body/tail	UR = 61	5-FU + Cisplatin + EBRT	ORR = 21%	11 month (nonresponders)	28 month
Brown KM 2008 78	Head/body/tail	BR = 13	Chemo + EBRT (chemo):	ORR-100%	NS	NS
			5FU (*n* = 3), Gemcitabine (*n* = 9), Capecitabine + Bevacizumab (*n* = 1)	R0 = 85%		
Allendorf JD 2008 79	Head/body/tail	UR = 78 (preop CRT) versus R = 167 (upfront resection)	Gem + Taxotere + Xeloda (81% of pts)	ORR = 76%	16.6 month upfront resection	17.7 month resected sp CRT
			75% pts also got EBRT	R0 = 84.7% of resected		
Golcher H 2008 80	Head/body	UR = 121 (preop CRT)	(5FU + Mitomycin) or (Gemcitabine + Cisplatin) + EBRT	ORR = 17%	21 month upfront resection	54 month resected sp CRT
		versus R = 58 (upfront resection)		R0 = 90% of 17%		
Turrini O 2009 81	Head	BR = 49, UR = 15	5-FU + Cisplatin + EBRT	ORR = 14%	13 month (UR), 14 month (all)	24 month
Stokes JB 2011 82	Head/body/tail	BR = 34	Capecitabine + EBRT	ORR = 46%	NS	23 month
				R0 = 75% of 46%		
Patel M 2011 83	Head/body/tail	BR = 17	Gemcitabine + Taxotere + Capecitabine → 5-FU + IMRT	ORR = 53%	15.6 month (all)	NS
				R0 = 89% of 53%		
Arvold ND 2012 22	Head/body/tail	BR = 24	5FU or Capecitabine + EBRT	PR = 30%	13.2 mo	19.4 month
		UR = 46		R0 = 79% of 20%		
Sho M 2012 84	NR	R = 61	Gemcitabine + EBRT	R0 92% versus 52% controls	28 month (neoadjuvant)	NS
		BR = 71				
Strobel O 2012 23	Head/body/tail	R = 120	(5FU, Gemcitabine, or Cetuximab-based) Chemoradiation or Chemotherapy	R0 35%, R1 50.8%, R2 13.3% of 46.7% resected	9 month	13 month
		UR = 137				
Boone BA 2013 25	Head/body/tail	BR = 12	FOLFIRINOX + EBRT	R0 = 33%	NS	NS
		UR = 13				
Faris JE 2013 26	Head/uncinated/tail	UR = 22	FOLFIRINOX +/− 5FU or Capecitabine + EBRT	PR = 27.3%	NS	NS
				SD = 72.7%		
				R0 = 23%		
Papavasiliou P 2014 24	Head/uncinate	NS	Gemcitabine or 5FU + EBRT	RR = NS	22 month (all)	NS
				R0 = 68.5%, R1 = 30.6%, R2 = 0.9%		
Rose JB 2014 32	Head/body/tail	BR = 64	Gemcitabine + Docetaxel	R0 = 87% of 61%	15.4 month	NR
					23.6 month (all)	

R, resectable; BR, borderline resectable; UR, unresectable; UNK, unknown; ORR, overall resection rate; R0, complete microscopically negative; R1, positive margin; PD, pancreaticoduodenectomy; EBRT, external beam radiotherapy; OS, overall survival; NS, not specified; NR, not reached; NA, not applicable; 5-FU, 5-Fluorouracil; FOLFIRINOX, Folinic acid, 5-Fluorouracil, Irinotecan, Oxaliplatin; C, cycle; →, followed by.

Given the risk of incomplete resection, neoadjuvant chemotherapy may similarly improve outcomes in borderline-resectable pancreatic cancer. In a retrospective report from Massachusetts General Hospital, 46 patients with unresectable and 24 patients with borderline-resectable disease were treated with neoadjuvant fluoropyrimidine-based chemoRT. Approximately 30 of these patients additionally received gemcitabine-based chemotherapy sequenced before the chemoRT. Compared with chemoRT alone, the use of neoadjuvant chemotherapy before chemoRT achieved improved median overall (18.7 vs. 12.4 months; *P* = 0.02) and progression-free survival (11.4 vs. 6.7 months; *P* = 0.02) 22.

The development of novel targeted or more contemporary cytotoxic therapeutics in metastatic pancreatic cancer was also investigated in the neoadjuvant setting. In a retrospective large single center study by Strobel and colleagues, 257 patients received neoadjuvant chemotherapy or chemoradiation 23. Only 120 (46.7%) underwent successful resection. Median postoperative survival was highest at R0 resected patients (24.6 months) compared to R1 (11.9 months) and R2 (8.9 months) demonstrating that margin status at surgery is still a major determinant of outcome, even with contemporary neoadjuvant therapy. The incorporation of FOLFIRINOX chemotherapy in the neoadjuvant setting has been explored in relatively small pilot studies. Boone and colleagues at the University of Pittsburgh treated 21 unresectable and borderline-resectable patients with this regimen. Two patients (9%) could not tolerate treatment and another three (14%) had disease progression. Overall, seven patients ultimately underwent resection of which 2 (10%) were initially unresectable and were felt to have been converted. Five (24%) of the treated and resected patients had significant histopathological response 24. Massachusetts General investigators treated 22 locally advanced pancreatic cancer patients in this manner with five of 22 patients achieving R0 resections after completing FOLFIRINOX, 5FU-based chemoradiation and surgical resection. However, three had distant recurrence and toxicity was significant with this approach 25.

While it is clear from the published studies that neoadjuvant therapy for pancreatic cancer appears feasible, the demonstrated benefits have been inconsistent. One of the primary limitations of these studies has been the use of historical controls as a comparison group. Over the intervening years, imaging technology has become increasingly accurate in delineating vessel involvement by pancreatic cancer, which is a major barrier to successful surgical resection. Indeed, such stage migration will, by definition, improve the apparent survival of patients newly diagnosed with both resectable as well as locally advanced pancreatic cancer. Similarly, advances in surgical techniques with more sophisticated vascular reconstruction capabilities, have also impacted the ability to obtain complete resections. However, it is not clear if complete resection in these borderline or previously unresectable patients with the use of modern vascular reconstruction techniques affords a similar long-term benefit as a complete resection in an initially clearly resectable patient. Another limitation includes an evolving definition of resectable disease. Surgical perspective by the treating physician adds a nongeneralizable bias to patient selection in regards to generating a homogenous treatment group as well as appropriate control matching. Only recently has a consistent definition been applied to studies, thus allowing cross-study comparisons. The summary data of neoadjuvant treatments (Table2) inventories the outcomes of patients organized by resectable, borderline resectable, and unresectable disease.

Further confounding the response of neoadjuvant treatment was a publication by the MD Anderson group. These authors reported that routine imaging does not reflect anatomic-pathologic changes associated with the effectiveness of neoadjuvant therapy 26. This retrospective study reviewed 122 patients with borderline-resectable pancreatic adenocarcinoma who had restaging of their disease after neoadjuvant treatment. Even though only 12% of patients met the RECIST imaging criteria for a partial response and only one patient (0.8%) was officially downstaged to resectable, (69% had stable disease and 19% had progressive disease), 66% of the patients were able to undergo pancreaticoduodenectomy. Median OS for patients who underwent surgery was 33 versus 12 months for those who did not.

In an attempt to determine aggregate outcome measures; Gillen et al. performed a systematic review on neoadjuvant therapy trials in pancreatic cancer 27. This meta-analysis looked at more than 100 neoadjuvant trials published since 1980, despite the heterogeneity of patients enrolled and regimens used. Of those patients considered resectable at diagnosis, approximately 74% went on to have surgical resection after neoadjuvant treatment with an R0 resection rate of 82%. Median survival in this group was 23.3 months (range 12–54 months) with 2-year survival of 47%. These survival results are comparable to patients who had initial surgery first followed by adjuvant therapy. Among the patients that were deemed to be initially unresectable, the overall resection rate after neoadjuvant treatment was 33% with R0 resection rate of 79%. The median OS was 20.5 months (range 9–62 months) with a 2-year survival of 50% for this group. Median survival, however, was only 10.2 months for those patients whose disease remained unresectable despite neoadjuvant treatment, which is similar to patients who were treated with palliative intentions (median survival 8–12 months). Despite the data, no standard regimen or sequence of treatment could be conclusively determined.

Further adding to the published data on this topic, Artinyan and colleagues conducted a population-based cohort series 28. Using the California Cancer Surveillance Program, 458 patients with pancreatic adenocarcinoma who underwent surgical resection and received systemic chemotherapy between 1987 and 2006 were retrospectively analyzed. Neoadjuvant treatment was delivered in about 9% of the patients and adjuvant treatment given in 91% cases. Patient characteristics such as age, gender, and tumor size were similar between the two groups; however, data on performance status or co-morbidities were not reported. There was a significantly lower rate of positive pathologic lymph nodes in the neoadjuvant group (45% vs. 65%) despite a higher rate of extra-pancreatic tumor extension. The neoadjuvant group also had significantly better OS compared with the adjuvant group (median survival, 34 vs. 19 months). While there are obvious limitations of a population-based cohort study, it is clear that only a small percentage of patients in that clinical practice environment received neoadjuvant treatment. Thus, such patients are likely highly selected individuals.

Nonetheless, this summative data combined with the systematic review and meta-analyses suggest that neoadjuvant therapy can be conducted safely in select patients and may possibly benefit a subset of those with resectable and borderline-resectable disease. By introducing early systemic treatment to combat distant relapses coupled with avoidance of a radical surgical resection in patients whose disease is biologically aggressive, neoadjuvant treatment offers many advantages. However, the magnitude of the impact has yet to be demonstrated or validated in a randomized controlled trial.

## Final Thoughts

Despite the opportunity to improve survival by incorporating systemic treatment in the neoadjuvant setting and better selection of patients with truly localized cancer, survival in this dreaded disease still remains modest. The key to significantly impacting survival, short of prevention, would be the identification of therapeutic interventions tailored to the patient, which can overcome the inherent resistance mechanisms evoked by the cancer. To achieve this goal of appropriate patient selection based on patient and disease characteristics and to optimize their chance of receiving the optimal medical, radiation and surgical treatment, a multidisciplinary, collaborative approach to the care of each and every patient with pancreatic adenocarcinoma is imperative. Despite all the strides in various oncologic disciplines the ability to offer a cure to most patients remains unachievable. Perhaps the best case for neoadjuvant multidisciplinary approach is to rapidly test novel hypotheses and the effects of various treatments on the tumor and the surrounding microenvironment. A neoadjuvant platform could gain insights into the tumor biology, which may ultimately hold the key to achieving cure for most, if not all patients afflicted with this deadly disease. However, this requires adequate tissue acquisition of the tumor tissue to confirm the initial diagnosis. Increased cytologic yield through endoscopically obtained core biopsies or circulating tumor cells will be required in the future to fully realize the molecular characterization and personalized therapeutics potential.

The less than ideal response to cytotoxic and targeted therapies is evident in the abundant trials in the metastatic setting that have consistently failed to demonstrate a statistically significant or clinically meaningful advantage over single agent gemcitabine (Table3). Only recently have FOLFIRINOX and gemcitabine with nab-paclitaxel raised the bar 29–31. While the use of two or three cytotoxic drugs showed significant survival advantage over gemcitabine alone (median OS 11.1 vs. 6.8 months; *P* < 0.001) in patients with metastatic disease, toxicities limit use to select patients with excellent performance status and no major comorbidities.

**Table 3:** Outcomes of selected randomized controlled clinical trials in metastatic pancreatic adenocarcinoma

Reference	Treatment	Total *N*	Median survival (month)	*P*-value
Bramhall SR, BJC 2002 85	Gemcitabine +/− Marimastat	239	5.4 versus 5.5	0.95
Berlin JD, JCO 2002 86	Gemcitabine +/− 5-FU	322	5.7 versus 6.5	0.09
Colucci G, Cancer 2002 87	Gem versus Gem + Cisplatin	107	5 versus 7.5	0.43
Rocha Lima CM, JCO 2004 88	Gemcitabine +/− Irinotecan	342	6.3 versus 6.6	0.78
Van Custem E, JCO 2004 89	Gemcitabine +/− Tipifarnib	688	6.1 versus 6.4	0.75
Louvet C, JCO 2005 90	Gemcitabine +/− Oxaliplatin	313	7.1 versus 9	0.13
Oettle H, Ann Oncol 2005 91	Gemcitabine +/− Premetexed	565	6.3 versus 6.2	0.84
Abou-Alfa GK, JCO 2006 92	Gemcitabine +/− Exatecan	349	6.2 versus 6.7	0.52
Heinemann V, JCO 2006 93	Gem versus Gem+Cisplatin	195	6 versus 7	0.15
Stathopoulous GP, BJC 2006 94	Gemcitabine +/− Irinotecan	145	6.4 versus 6.5	0.97
Herrmann R, JCO 2007 95	Gemcitabine +/− Capecitabine	319	7.2 versus 8.4	0.23
Moore MJ, JCO 2007 96	Gemcitabine +/− Erlotinib	569	5.9 versus 6.3	**0.03**
Poplin E, JCO 2009 97	Gemcitabine versus fixed dose rate Gemcitabine versus Gemcitabine + Oxaliplatin	832	4.9 versus 6 versus 5.7	**0.04**
				0.22
Van Custem E, JCO 2009 98	Gemcitabine+Erlotinib +/− Bevacizumab	301	6.0 versus 7.1	0.20
Kindler HL, JCO 2010 99	Gemcitabine +/− Bevacizumab	602	5.9 versus 5.8	0.95
Philip PA, JCO 2010 100	Gemcitabine +/− Cetuximab	745	5.9 versus 6.3	0.23
Conroy T, NEJM 2011 24	Gemcitabine versus FOLFIRINOX	342	6.8 versus 11.1	**<0.001**
Von Hoff, NEJM 2013 101	Gemcitabine +/− nab-paclitaxel	861	8.5 versus 6.7	***P***** < 0.001**

5-FU, 5-fluorouracil; +/−, one arm with and one arm without the drug following the sign.

Bold indicates statistically significant.

Thus, the future of effective pancreatic cancer therapy must take into consideration not just the cancer, but also the interplay of the tumor microenvironment, the inherent biologic features that confer early metastatic potential to the cancer, the role of cancer stem cells in therapy resistance mechanisms, and novel gain of function mutations that may serve as new targets for therapeutic disruption. Only an adequately powered prospective study will be able to determine if neoadjuvant therapy provides a survival advantage for early stage pancreatic cancer patients. This study should randomize patients with borderline-resectable disease to the most effective systemic chemotherapy and/or chemoradiotherapy before or after surgery, and explore important outcomes such as relapse free survival and OS. Secondary end points should include resection rates, toxicity and surgical complications. At this moment, however, no such trial exists and neoadjuvant therapy should be conducted as part of an investigational program.

## Conflict of Interest

None declared.
